# The Role of Structure in the Biology of Interferon Signaling

**DOI:** 10.3389/fimmu.2020.606489

**Published:** 2020-11-12

**Authors:** Mark R. Walter

**Affiliations:** Department of Microbiology, University of Alabama at Birmingham, Birmingham, AL, United States

**Keywords:** interferon, IFN, type-I, type-II, type-III, receptor complex, IFN signaling, structure

## Abstract

Interferons (IFNs) are a family of cytokines with the unique ability to induce cell intrinsic programs that enhance resistance to viral infection. Induction of an antiviral state at the cell, tissue, organ, and organismal level is performed by three distinct IFN families, designated as Type-I, Type-II, and Type-III IFNs. Overall, there are 21 human IFNs, (16 type-I, 12 IFNαs, IFNβ, IFNϵ, IFNκ, and IFNω; 1 type-II, IFNγ; and 4 type-III, IFNλ1, IFNλ2, IFNλ3, and IFNλ4), that induce pleotropic cellular activities essential for innate and adaptive immune responses against virus and other pathogens. IFN signaling is initiated by binding to distinct heterodimeric receptor complexes. The three-dimensional structures of the type-I (IFNα/IFNAR1/IFNAR2), type-II (IFNγ/IFNGR1/IFNGR2), and type-III (IFNλ3/IFNλR1/IL10R2) signaling complexes have been determined. Here, we highlight similar and unique features of the IFNs, their cell surface complexes and discuss their role in inducing downstream IFN signaling responses.

## Introduction

IFNs were discovered more than 60 years ago (1957) as substances that protect cells from viral infection (1, 2). Based on their sensitivity to pH, IFNs were designated as either type-I (pH stable) or type-II (pH sensitive) (2, 3). Characterization of their distinct amino acid sequences and crystal structures (4, 5) (6–8) further validated the classification of IFNα/β and IFNγ as type-I and type-II IFNs, respectively. The type-I family expanded (9) to include 12 IFNαs (10–13) encoded by 13 genes (IFNα1/13 encode the same protein), IFNβ, IFNϵ (14), IFNκ (15), and IFNω (16). Genome analysis in 2003 identified a new type-III IFN family (IFNλs) (17, 18), which by sequence and subsequent structure analysis (19) were similar to IL10 family cytokines (12, 20–22), in particular IL-22 (23, 24). With the discovery of IFNλ4 in 2013 (25), a total of 21 IFNs (**Table 1**) exhibit not only antiviral activity, but anti-tumor actions, and the ability to modulate the adaptive immune response.

**Table 1:** IFN families and their receptor complexes.

		High Affinity Receptor	Low Affinity Receptor	IFNs						
Type-I IFNs		**IFNAR2**	**IFNAR1**	IFNα1/13*,	IFNα2,	IFNα4,	IFNα5,	IFNα6,	IFNα7,	IFNα8,
				IFNα10,	IFNα14,	IFNα16,	IFNα17,	IFNα21,	IFNβ,	IFNϵ,
				IFNκ,	IFNω					
		JAK1	TYK2							
Type-II IFNs		**IFNGR1**	**IFNGR2**	IFNγ						
		JAK1	JAK2							
Type-III IFNs		**IFNLR1**	** **	IFNλ1,	IFNλ2,	IFNλ3,	IFNλ4			
		** **	**IL10R2**	IFNλ1,	IFNλ2,	IFNλ3,	IFNλ4,	IL10,	IL22,	IL26
		JAK1	TYK2							

*IFNα1/13 encode the same amino acid sequence [(see 9)].

The pleotropic biological activities of the three IFN families are initiated by binding and subsequent assembly of heterodimeric receptor complexes on the cell membrane (**Table 1**). The 16 type-I IFNs bind and signal through the IFNAR1 and IFNAR2 receptor complex, type-II IFNγ binds to IFNGR1 and IFNGR2 chains, and the type-III IFNs signal through IFNλR1 and IL-10R2 receptor chains. Each receptor heterodimer consists of a high affinity receptor chain (e.g., IFNAR2, IFNGR1, IFNλR1) and a low IFN affinity receptor chain (IFNAR1, IFNGR2, IL10R2). The high and low affinity receptors exhibit nM and µM/mM affinity, respectively, for their cognate IFNs (26–30). Despite variable affinities, the high and low affinity type-I and type-II receptors are specific for their cognate IFN family members. In contrast, IFNλR1 is specific for type-III IFNλ family members, but the low affinity IL-10R2 chain is a shared receptor that also participates in IL10, IL22, and IL26 signaling complexes (12, 31–33).

IFN receptor complex formation activates Janus kinases (JAKs) that initiate IFN-mediated intracellular signaling cascades (34–38). The JAKs constitutively associate with the intracellular domains (ICDs) of the IFN receptors through non-covalent interactions (**Table 1**). Type-I and type-III IFN receptors use the same JAKs for signal transduction. The high affinity IFNAR2 and IFNλR1 receptors associate with JAK1, while low affinity IFNAR1 and IL10R2 associate with TYK2. In contrast, type-II IFNGR1 and IFNGR2 associate with JAK1 and JAK2, respectively (39, 40). The ICDs of the low affinity receptors are 69–100 amino acids long and their main purpose appears to be to bind their respective kinases for activation upon receptor complex formation. The high affinity receptor ICDs range from 223 to 271 amino acids in length and contain multiple tyrosine residues that upon phosphorylation by the JAKs, recruit STATs that become phosphorylated themselves, and translocate to the nucleus where they activate interferon-stimulated genes (ISGs) (40, 41). In addition to using the same JAKs, type-I and type-III IFNs induce the same STAT1/STAT2/IRF9, ISGF3 transcription complex (40–42). IFNγ activates phospho-STAT1 homodimers, but not ISGF3, which is reflected in the ~1,000-fold lower anti-viral activity of IFNγ compared to the type-I and type-III IFNs (43, 44). In addition to activating distinct intracellular signaling pathways, type-I/III IFNs are produced in cells upon viral infection, or infection by other pathogens, through pattern recognition receptor pathways, including RIGI, MDA7, PKR, TLR3, TLR7, TLR9, and STING (40, 45–48). In contrast, type-II IFNγ is produced predominantly by antigen-activated T lymphocytes (39). Thus, type-I/III IFNs are products of innate immune system, designed to establish direct and immediate antiviral states in cells, yet can also modulate adaptive immune responses. Type-II IFNγ is itself a product of adaptive immunity that acts on cells of innate immunity, notably macrophages. As a potent macrophage activator, IFNγ is essential for combating mycobacteria and other intracellular pathogens (49, 50). IFNGR1 deficiencies in individuals are associated with mycobacterial infections, while individuals with IFNAR2, or IFNAR1, deficiencies have had life threatening illness following vaccination with mumps, measles, and rubella (MMR) vaccines (51, 52). Together, these data highlight the distinct roles of these IFNs in controlling different pathogens.

While there is only one IFNγ, it is remarkable that humans encode 16 different type-I and 4 type-III IFNs that induce the same fundamental ISGF3-mediated anti-viral program in cells (17, 18, 53, 54). The necessity of this remarkable arsenal of IFNs to combat virus, and other pathogens (55–58), remains an area of intense investigation. Given the complexity of IFN signaling, this review describes the fundamental structural organization of each IFN receptor complex in generating IFN signaling responses. The main emphasis is to define how structure impacts IFN-IFN receptor affinity, specificity, and the role of the overall architecture of the complex to position receptor ICDs for intracellular JAK/STAT activation and subsequent cellular activity.

### Structures of the Type-I, Type-II, and Type-III IFNs

**Figure 1:**
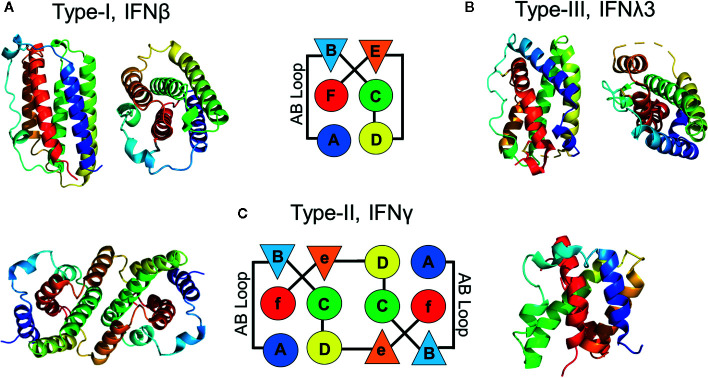
Structures of IFN family members. Schematic, and ribbon diagrams, show the six secondary structural elements of the type-I **(A)**, pdbid = 1AU, type-III **(B)**, pdbid = 3HHC, and type-II **(C)**, pdbid = 6E3K IFNs. IFN structures are rainbow colored from the N-terminus helix A (blue) to the C-terminal helix F (red).

All IFNs adopt α-helical structures with unique up-up-down-down topology (21), relative to other α-helix bundle proteins (**Figure 1**). Each IFN consists of six secondary structural elements, denoted A-F, of which helices A, C, D, and F form an anti-parallel four helix bundle. Loop elements B and E exhibit more variable secondary structures, ranging from additional helices to extended segments that pack against the edge of the four-helix bundle (helices A, C, D, and F). The α-helices of the Type-I IFNs are long, straight, and essentially parallel to one another (**Figure 1A**). Despite considerable sequence diversity (35%–95%), all 16 IFNs adopt the same α-helical structure (4, 5, 59–63). In contrast to type-I IFNs, type-III IFNs are comprised of shorter helices that contain several kinks, which form a more compact bundle (**Figure 1B**). As a result, type-III IFNs adopt structures that are more similar to the IL-10 family cytokine IL-22 than to type-I IFNs (12, 19, 23, 24, 64). This is interesting from a functional perspective since IL-22 induces anti-bacterial activity in the gut and skin through a tissue-restricted receptor complex of IL22R1 and IL10R2 (22, 32, 65–70). Thus, IFNλs and IL-22 control viral and bacterial challenges, respectively, at barrier surfaces (22, 64, 71). As a “mucosal IFN”, IFNλs have been promoted as an optimal drug to treat respiratory viruses, such as Severe Acute Respiratory Syndrome Coronavirus 2 (SARS-CoV-2), which causes COVID-19 (72). However, IFNλ signaling in mice prevents lung epithelial repair, leading to bacterial superinfections (73, 74). Other studies suggest type-I IFNs, not IFNλs, might be most efficacious and safe in treating SARS-CoV-2 (75). Overall, these studies highlight the complexity of IFN signaling at barrier surfaces and differences in IFN signaling outcomes in mice vs. humans.

In contrast to the monomeric type- I and type-III IFNs, IFNγ adopts an intercalated dimer structure, where helices E and F from one chain are “swapped” with the other subunit of the dimer (**Figure 1C**). Like the IFNλs, the structure of IFNγ is most similar to IL10, which is the founding member of the IL-10 cytokine family (12, 21, 32, 76–78). These data confirm that each IFN family adopts a distinct α-helical scaffold, which must “handle” various amounts of sequence variation to regulate engagement of their cellular receptors. For example, there is one highly conserved type-II IFNγ dimer, whereas there are 16 monomeric type-I IFNs (35%–95% sequence identity) and 4 type-III IFNs (28%–96% sequence identity) that exhibit variable amino acid sequence identities. This highlights the distinct mechanisms used by each IFN family to regulate biological activity. Receptor homodimerization by IFNγ, versus variable IFN/IFN-receptor contacts by monomeric type-I and type-III IFNs. These mechanisms will be reviewed in more detail below.

### The Type-III IFNλ/IFNλR1/IL10R2 Complex

**Figure 2:**
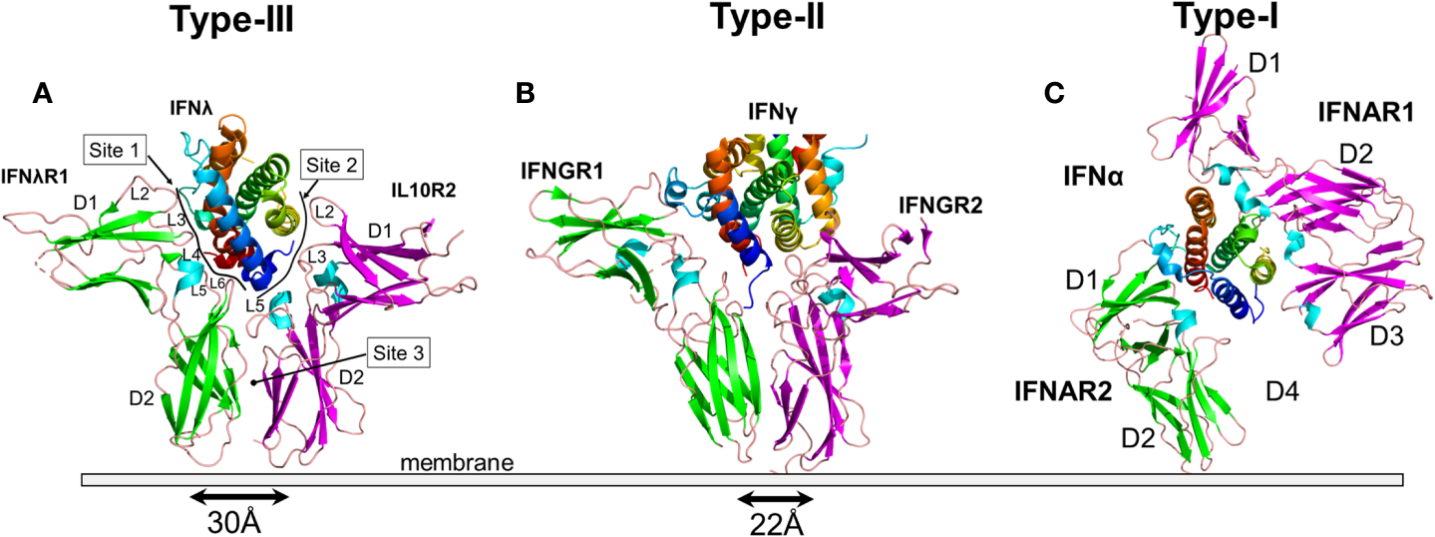
Structures of the IFN Receptor Complexes. Ribbon diagrams of the type-III **(A)**, pdbid = 5T5W, type-II **(B)**, pdbid = 6E3K, and type-I **(C)**, pdbid = 3SE4, receptor complexes. IFNs are rainbow colored as described in Figure 1. The β-strands of the high affinity receptor chains are colored green and low affinity chains are colored magenta. For the type-II IFNγ receptor complex, only one IFNγ subunit is shown to emphasize the similarity of “half” of the complex with the type-III IFN receptor complex. The separation of the C-termini of the type-III IFNλR1/IL-10R2 and type-II IFNGR1/IFNGR2 receptor chains, where they enter the membrane are 30Å and 22Å, respectively. A D2-D4 interaction was not observed in structures of the IFN/IFNAR1/IFNAR2 complex.

**Figure 3:**
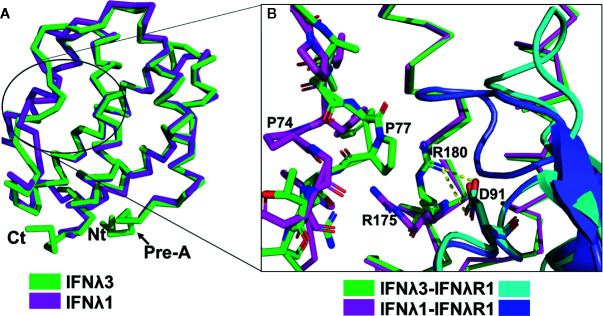
Subtle structural changes between IFNλ1/IFNλ3 alter IFNλR1 Contacts. **(A)** Alpha carbon diagram of the superposition of IFNλ1 and IFNλ3. The location of structural differences in the B loop regions of IFNλ1 and IFNλ3, as discussed in the text, are circled. **(B)** Enlargement of the B loop “proline flip” observed in IFNλ1 and IFNλ3 structures and its influence on the conformation of Arg-180^IFNλ3^ (green), where it makes a salt bridge with IFNλR1 Asp-91. In contrast, IFNλ1 Arg-175 (magenta) extends away from IFNλR1 Asp-91 towards the B loop.

The type-III IFNλ receptor complex (79) exhibits the simplest architecture of the three IFN families. Monomeric IFNλs assemble 1:1:1 signaling complexes with high affinity IFNλR1 and low affinity IL10R2 receptors (**Figure 2A**). IFNλR1 and IL10R2 both consist of two β-sandwich domains (D1, D2), where the D2 domains are positioned closest to the membrane. IFNλR1 binds to the IFNλs using five receptor loops (L2-L6) that are located at the junction of the D1 and D2 domains. The IFNλR1 binding loops contact IFNλ residues located on helix A, the AB loop, and helix F. Although differing in detail, the high affinity IFNλ/IFNλR1 site-1 binding site is conserved with type-I and type-II high affinity receptor complexes (**Figure 2**). The low affinity IL10R2 binding site-2 consists of N-terminal IFNλ residues, prior to the start of helix A (e.g., the pre-A region (80), also see **Figure 3A**), residues on helix C, and on the segment of helix D that runs parallel to the pre-A region. IL10R2 uses a subset of the same loops used by IFNλR1 (loops L2, L3, and L5) to contact IFNλ. Thus, the IFNλ-IL10R2 site-2 interface is discontinuous, making a smaller L2/helix D contact (site-2a) and a larger interaction between L3/L5 and IFNλ pre-A and helix D (Site 2b).

In addition to IFNλ-IL10R2 site-2 contacts, IL10R2 forms an additional D2-D2 site-3 interface with IFNλR1. Thus, the complete IL10R2 binding site is only formed once IFNλ binds to IFNλR1. This structural organization ensures IFNλ receptor complex formation is cooperative, where the IFNλ/IFNλR1 complex forms first, followed by binding of IL10R2 to site-2 and site-3. Once formed, the assembled IFNλ complex positions the C-terminal ends of IFNλR1 and IL10R2 30Å apart from one another, prior to entering the membrane. The combined site-2 and site-3 interfaces bury over 1,500Å (2) of surface area, which is more than twice the surface area buried in the high affinity IFNλ3/IFNλR1 site-1 interaction. However, despite this extensive interface, there are few energetically critical interactions. Thus, the affinity of IL-10R2 for the IFNλ3/IFNλR1 complex (e.g., site-2 + site-3) is 15 µM (79), which is ~15× lower than the affinity of IFNAR1 for most IFN subtypes (26, 27). While IFNλ3/IFNλR1 represents the “high affinity” interaction in the complex, the measured *K*D of 850nM (79) is ~1 log lower than the affinity of the weakest type-I IFN for IFNAR2 (e.g., IFNα1, *K*D ~100nM).

Due to the low affinity of the IFNλs for their receptors, the IFNλs are sensitive to the expression levels of their receptors on cells. In fact, a major distinction between type-I and type-III IFNs is the unique distribution of their receptors on different cell types (81, 82). Type-I IFNAR1 and IFNAR2 receptors are present on all nucleated cells, while IFNλR1 expression is predominantly limited to epithelial cells, as mentioned for IL22R1 earlier (22, 70). Thus, IFNλ signaling appears to be specialized for combating viral infections at epithelial barrier surfaces such as the lung, gut, and liver (83). This has most impressively been shown by demonstrating IFNλ, but not type-I IFN, is essential for controlling norovirus infection (84). Although gut epithelial cells in this study express type-I IFNARs, their expression is limited to the apical surface of the cells, and no IFNAR expression is observed on the basolateral surface. Thus, the selective signaling of IFNλ in gut epithelial cells was only fully appreciated within the organization of the intact gut in animals. While IFNλ activity appears “weak” in many cell-based assays, *in vivo* data suggests potent IFNλ signaling in the context of tissues and organs. It should be noted that type-I IFNs, IFNϵ and IFNκ, protect the female reproductive track (85–87) and skin (15, 88), respectively. Notably, like the IFNλs, IFNϵ and IFNκ exhibit “low” affinity for the type-I receptors, relative to most type-I IFNs (89).

### Insights From IFNλ1/IFNλR1 and IFNλ3/IFNλR1 Binary Structures

Both IFNλ1/IFNλR1 and IFNλ3/IFNλR1 binary complex structures have been solved (79, 90). IFNλ1 and IFNλ3 adopt very similar structures, with a root-mean-square deviation (r.m.s.d.) of 0.6Å. Similarly, IFNλR1 binding to either IFNλ1 or IFNλ3 exhibits an r.m.s.d. of 0.68Å. Finally, the structure of unbound IL10R2 (91) and IL10R2 bound to IFNλ3 exhibit an r.m.s.d. of 1.3Å. The larger r.m.s.d. is due to changes in the conformation of the IL10R2 L5 binding loop upon contacting IFNλ3. Despite this difference, the overall structures of bound and unbound IL10R2 are the same. These structural comparisons suggest all IFNλs assemble a signaling complex with the same overall architecture. Thus, IFNλ biological potency is not regulated by the structure of the ternary complex, but by the affinity of each IFNλ for the IFNλR1 and IL10R2 chains, and ultimately the stability of the complex.

*In vitro* cell-based assays demonstrate IFNλ3 exhibits twofold greater antiviral potency than IFNλ1 (92). Although a detailed analysis of IFNλ receptor binding affinity has not been completed, we expect the IFNλ3/IFNλR1 complex should exhibit differences from the IFNλ1/IFNλR1 complex, consistent with a higher affinity interaction. Comparison of IFNλ1 and IFNλ3 structures (**Figure 3A**) reveals the B loop regions of IFNλ1 and IFNλ3 exhibit different conformations, particularly Pro-74^IFNλ1^/Pro-77^IFNλ3^ (**Figure 3B**). In IFNλ3, Pro-77 moves in toward helix F, while in IFNλ1 Pro-74 moves away from helix F. This “proline flip” alters the position of the conserved Arg-175^IFNλ1^/Arg-180^IFNλ3^, located on helix F (**Figure 3B**). In IFNλ3, the guanidino group of Arg-180 packs against Pro-77, which positions it for a bivalent salt bridge with IFNλR1 residue Asp-91. A series of IFNλ3 alanine mutants were tested for antiviral activity and identified Phe-179 as the most important IFNλ3 residue for inducing antiviral activity (19). Since IFNλ3 Phe-179 is adjacent to Arg-180, it is likely that mutation of Phe-179 to an alanine disrupts the Arg-180^IFNλ3^/Asp-91^IFNλR1^ salt bridge, which reduces IFNλR1 binding affinity and antiviral activity.

The “proline flip” observed between IFNλ1 and IFNλ3 (**Figure 3B**) may also provide mechanistic insight into the reduced biological activity of the IFNλ4 single nucleotide polymorphism (SNP), rs117648444. Rs11768444 corresponds to IFNλ4-Pro70Ser, which exhibits reduced antiviral activity, relative to wildtype IFNλ4 (25, 93). Understanding IFNλ4 SNPs is important since several groups have mapped the major genetic determinant of hepatitis C virus (HCV) clearance, in response to treatment with IFN-α plus ribavirin, to the type-III IFN loci (94–96). Ultimately, IFNλ4 activity has been implicated as the causative agent of HCV clearance failure in patients that encode “active” IFNλ4 protein, as opposed to inactive IFNλ4 protein (25). Despite sharing ~28% sequence identity with IFNλ3, IFNλ4 adopts the same α-helical fold as other IFNλs and binds to IFNλR1 and IL10R2 (97). Amino acid sequence alignments show IFNλ4 Pro-70 is identical to IFNλ3 Pro-77, suggesting the IFNλ4 Pro70Ser mutation impacts IFNλ4-IFNλR1 interactions by altering the structure of IFNλ4 Arg-163, as described for Arg-180 in IFNλ3 (**Figure 3B**).

IFNλ2 has not been studied to the same extent as the other IFNλs, presumably because it was shown to exhibit ~5–10× lower antiviral activity (53, 98). The IFNλ2 amino acid sequence differs from IFNλ3 by only 6 amino acids. Modeling the structure of IFNλ2 based on the structure of IFNλ3 suggests, R28H occurs in a non-structured region at the N-terminus of the molecule, where it is not predicted to alter receptor binding. K70R and R72H are located in the AB loop of IFNλ2, but do not contact IFNλR1. Furthermore, an IFNλ3 R72A mutant reduced IFNλ3 anti-viral activity by only 30%, suggesting these residue changes cannot explain the lower activity of IFNλ2. Residues V92M and H156Y are located on exposed surfaces of IFNλ2 helices C and E, respectively, which are located opposite the IFNλR1 and IL10R2 binding sites. Thus, if these amino acids were responsible for the lower activity of IFNλ2, this would support the hypothesis of some groups that IFNλ may bind to another, unidentified, receptor chain (83). Finally, L133F is located on helix D, where the sidechain is buried in the hydrophobic core of IFNλ2. The L-to-F amino acid change cannot be incorporated into the hydrophobic core of the IFNλ3 structure without distorting helices A, D, or F. This suggests L133F may be the main residue responsible for the reduced biological activity of IFNλ2, relative to IFNλ3.

### The Type-II IFNγ/IFNGR1/IFNGR2 Complex

**Figure 4:**
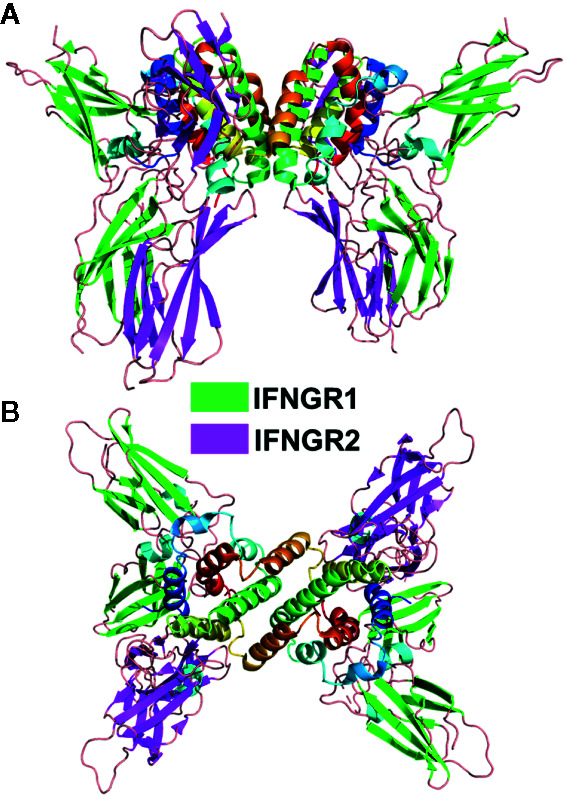
Dimeric IFNγ/IFNGR1/IFNGR2 Complex. Ribbon diagram of the 1:2:2 IFNγ dimer/IFNGR1/IFNGR2 complex (pdbid = 6E3K). Two views of the complex are shown. The first is approximately perpendicular to the IFNγ twofold axis **(A)** and the second is parallel to the twofold axis **(B)**.

The type-II IFNγ receptor complex provides an important structure to further understand the type-I and type-III complexes (99). The unique intercalated dimer structure (6) of IFNγ distinguishes it from the disulfide-linked monomeric type-I and type-III IFNs (4, 19, 100). The IFNγ dimer assembles a symmetric 1:2:2 IFNGR1/IFNGR2 heterodimeric complex (99, 101) (**Figure 4**), compared to the 1:1:1 heterodimeric complexes of the type-I and type-III IFNs (**Figure 2**). In the dimeric complex, the twofold-related C-termini of the IFNGR1/IFNGR2 heterodimers are positioned 85Å apart from one another. As suggested from the analysis of the structurally related IL10 dimer (102), the dimeric IFNγ positions IFNGR1 and IFNGR2 (**Figure 4**), and their respective ICDs, in an optimal dimeric arrangement to recruit inactive STAT1 dimers (103) for subsequent phosphorylation and activation of STAT1 homodimers (104). Disruption of the dimeric IFNγ receptor complex architecture, using engineered monomeric IFNγs, which assembles ½ of the dimeric IFNγ/IFNG1/IFNGR2 (see **Figure 2** vs. **Figure 4**), drastically reduced some IFNγ-induced biological activities (7, 8, 99, 102, 105). Additional IFNγ mutants confirmed the dimeric arrangement of IFNGR1, not IFNGR2, was essential for full STAT1 phosphorylation (99). In contrast to STAT1, many additional pathways activated by IFNγ, including MAP kinase, PI3K, and CaMKII (106), appear not to be equally sensitive to IFNγ-mediated IFNGR1/IFNGR2 dimerization. Thus, at least on some cells, engineered IFNγ monomers can induce the same levels of cell surface HLA-A as the WT IFNγ dimer (99). Interestingly, it should be noted that neurons appear to naturally manipulate IFNγ signaling outcomes by maintaining low STAT1 levels, which results in potent IFNγ-mediated activation of ERK1/2 (107). Overall, the dimeric architecture of the IFNγ/IFNGR1/IFNGR2 complex is critical for inducing the full spectrum of IFNγ-mediated pleotropic activities (108), which includes macrophage activation (109, 110), tumor surveillance (111, 112), and protection from intracellular pathogens, including mycobacteria (50, 113).

Despite the larger dimeric assembly, within one IFNγ subunit, IFNGR1 and IFNGR2 form similar site-1, site-2, and D2-D2 site-3 interfaces, as previously described for the IFNλ/IFNλR1/IL10R2 complex (**Figure 2B**). Compared to IFNλ/IFNλR1, the IFNγ site-1 interface is more extensive with major contacts between the AB loop and helix F of IFNγ and IFNGR1 L2-L6 loops. The site-2 IFNγ/IFNGR2 interface is comprised almost exclusively of contacts with IFNγ helix D and no contacts with helix A, the main contact region in the IFNλ complex. Despite these differences, IFNGR2 still forms a D2-D2 site 3 interface with the IFNGR1, which positions the C-termini of the receptors 22Å apart at the cell surface prior to their entry into the membrane. Thus, assembly of the IFNγ signaling complex is cooperative, requiring the formation of the IFNγ/IFNGR1 binary complex first, followed by IFNGR2 binding to induce cell signaling.

### The Type-I IFN/IFNAR1/IFNAR2 Complex

The type-I IFN receptor complex is distinct from both the type-II and type-III receptor complexes (**Figure 2**). The high affinity IFNAR2 chain adopts a two-domain D1/D2 receptor structure, as observed for IFNλR1 and IFNGR1 chains (**Figure 2**) (114). NMR and X-ray structures confirm IFNAR2 binds to an IFN site-1 epitope that is comprised of residues on helix A, the AB loop, and helix F, similar to the type-II and type-III IFNs (100, 115, 116). IFNAR2 makes extensive interactions with Arg-33 (IFNα2 numbering) in the AB loop of the IFNs. Arg-33, and the structurally adjacent Leu-30, account for approximately two thirds of the IFNα2/IFNAR2 binding energy (29, 100, 117). Additional critical contacts occur with the IFNAR2 L3 and L4 binding loops, which contact helix F residues Met-148 and Arg-149 (IFNα2 numbers) (117). Although we know that all 16 IFNs exhibit a variety of affinities for IFNAR2 (26–28, 89), the mechanisms that control IFNAR2 affinity for each IFN subtype remains incomplete. In general, it appears that subtle changes to residues around these energetically critical residues modulate IFN-subtype IFNAR2 affinity.

The type-I IFN low affinity receptor chain, IFNAR1, is completely unique relative to the other IFN and IL10 family cytokine receptors (**Figure 2**). IFNAR1 consists of four β-sandwich domains (D1-D4), similar to tandem D1/D2 receptors, where the D4 domain is the membrane proximal domain. The D2 and D3 domains of the receptor form an extensive interface with one another, while the D1 domain can undergo rigid body movements. Overall, IFNAR1 D1-D3 domains form an IFN-binding module, while the D4 domain is attached to D3 by a flexible linker that allows the D4 domain to adopt multiple conformations, even when bound to IFN (100, 118). Despite a unique structure, IFNAR1 loops at the ends of D1, D2 and D3 domains contact IFN helices C, D, and E, with the D1 domain “closing down” on helix E, like a hand grabbing a glass.

Based on the features described above, the binding of type-I IFNs by IFNAR1 represents a novel protein recognition paradigm. First, the IFNAR1-IFN contact surface, consisting of IFN helices C, D, and E, is larger than for the other IFN complexes. Second, the membrane proximal D4 domain of IFNAR1 does not form a site 3 interface, at least not a stable interface, with the D2 domain of IFNAR2. This suggests that by increasing the size of the IFNAR1-IFN site-2 interface (see **Figure 2C**), using novel D1/helix E interactions, the type-I IFN complex no longer requires a site-3 interface. Thus, for the type-I IFN complex, there is no structure-based cooperativity enforced by a D2-D4 site-3 interaction. Rather, receptor complex assembly and stability is controlled completely by IFN-IFNAR2 and IFN-IFNAR1 affinities. While it is possible that free IFNs, and IFNs bound to IFNAR2, could exhibit different affinities for IFNAR1, resulting in an affinity-based cooperative binding mechanism, this has not been demonstrated experimentally.

**Figure 5:**
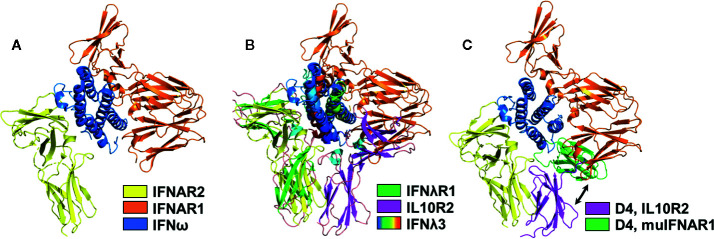
Structural Models of the IFNAR1 D4 Domain. **(A)** Ribbon diagram of the type-I IFN (IFNω, blue)/IFNAR1 (orange)/IFNAR2 (yellow) complex structure (pdbid = 3SE4), which lacks the IFNAR1 D4 domain. **(B)** Superposition of the IFNλ3 (rainbow)/IFNλR1 (green)/IL10R2 (magenta) ternary complex on the IFN/IFNAR1/IFNAR2 structure positions the IL10R2 D2 domain (magenta), such that it could represent the transient location of the IFNAR1 D4 domain forming an IFNAR2 D2-IFNAR1 D4 stem interaction. **(C)** A second possible location of the human IFNAR1 D4 domain is shown by superimposing the murine IFNβ/IFNAR1 complex (pdbid = 3WCY) on the IFN/IFNAR1/IFNAR2 complex. The position of the modeled D4 domain (green), derived from the murine IFNβ/IFNAR1 structure is shown in green, and the location of the IFNAR1 D4 domain obtained from superimposing the IFNλ receptor complex is shown in magenta. Since the human IFNAR1 D4 domain does not form a stable D2-D4 interaction with IFNAR2, D4 may transition between green and magenta conformations to induce biological activity. The exact role of the D4 domain in IFN signal transduction remains unknown.

The mechanistic role of the IFNAR1 D4 domain in type-I IFN receptor activation remains unclear since the D4 domain was not observed in crystal structures of the IFN/IFNAR1/IFNAR2 complex (**Figure 5A**). To identify possible location/s of the IFNAR1 D4 domain, the IFNλ3/IFNλR1/IL10R2 complex was superimposed onto the IFNω/IFNAR1/IFNAR2 complex (**Figure 5B**). In this model, the D1 domain of IL10R2 overlaps with the IFNAR1 D3 domain and the putative location of the IFNAR1 D4 domain, represented by the IL10R2 D2 domain, is adjacent to the IFNAR2 D2 domain creating a D2-D4 site-3 interface, as observed in type-II and type-III complexes (**Figure 2**). A second possible position of the D4 domain is provided by the structure of the murine IFNβ/IFNAR1 binary complex (119), where all four domains of IFNAR1 were observed. Superposition of the murine IFNβ/IFNAR1 complex on the IFN/IFNAR1/IFNAR2 human complex places the C-terminal ends of IFNAR2 D2 and IFNAR1 D4 51Å apart (**Figure 5C**), in contrast to 30Å and 22Å for the IFNλ and IFNγ complexes, respectively. These models lead to two possible conclusions. First, type-I IFNs assemble a novel “open” complex with the C-terminal ends of IFNAR1 and IFNAR2 separated by ~50Å. Second, the “open” conformation is an inactive complex, which must “close” to form a D2/D4 site-3 interface to induce IFN activity. Our analysis suggests that IFN binding to IFNAR2 and IFNAR1 promotes transient IFNAR2-D2/IFNAR1-D4 interactions. Thus, the stability of the IFN/IFNAR1/IFNAR2 interaction would control the number of transient “open”/”closed” D2-D4 site-3 binding events, which could influence signaling strength. Thus, the stability of the IFN/IFNAR2 and IFN/IFNAR1 interactions would regulate signaling, as has been previously described (120).

Despite structures that reveal extracellular IFN-receptor recognition and assembly mechanisms, there remain questions about IFN-mediated signal transducing events that initiate and sustain cellular activation. For example, it remains unclear how all 16 IFNs, that exhibit a spectrum of affinities for the IFNARs (weak/strong), can all activate a subset of genes associated with antiviral activity on all cells, while additional cellular functions of the IFNs, one such readout being anti-proliferative activity, correlates with IFN-IFNAR affinity (121). These two distinct cellular readouts, labeled as robust and tunable activation (121), might be explained by an IFNAR1/2 pre-association model (122) and an IFN-mediated IFNAR1/2 heterodimerization model (123), respectively. The IFNAR pre-association could account for rapid IFN-mediated activation of antiviral gene expression, while IFN-mediated IFNAR dimerization could account for tunable gene expression. The implication of the pre-association model is that the IFNs induce a structural change in the IFNARs that activate JAK1/TYK2 and induce rapid anti-viral gene expression, while the dimerization model relies solely on IFN-mediated dimerization of the IFNARs to activate JAK1/TYK2 and subsequently induce IFN-mediated gene expression. Technical issues, specifically analysis of artificially high IFNAR expression levels, have been suggested to be responsible for the observation of pre-associated IFNARs (123). Unfortunately, the investigators criticizing the pre-association model did not confirm that overexpression of the IFNARs leads to IFNAR1/2 interactions. Nonetheless, the cortical actin cellular meshwork and/or lipid rafts could provide a suitable mechanism to “concentrate” IFNARs for rapid induction of robust antiviral genes by all IFNs, while still allowing tunable activities that are dependent on IFN-IFNAR affinities (124). Overall, the data suggest that the major mechanism regulating IFN activation is IFN-mediated IFNAR1/2 heterodimerization, although some recent data suggests IFN-induced IFNAR conformational changes may also regulate IFN activity (125).

### The Murine Type-I IFN Family Is Distinct From Human Type-I IFNs

**Figure 6:**
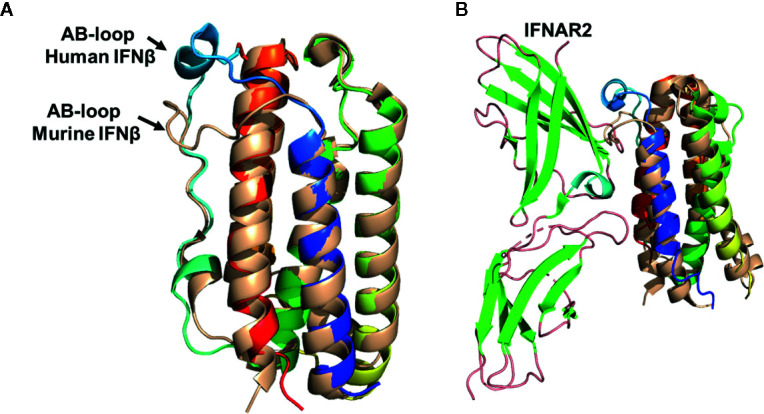
Structural Comparison of human and murine IFNβ. **(A)** Structural superposition of human IFNβ (colored as in **Figure 1**, pdbid = 1AU1) and murine IFNβ (wheat, pdbid = 1WU3), highlighting their distinct AB loop structures. **(B)** Structural superposition of murine and human IFNβ onto IFNα2 from the human IFNα2/IFNAR2 crystal structure. The resulting structural model results in steric clashes between the murine IFNβ AB loop and IFNAR2 binding loops, but not for the human IFNβ/IFNAR2 model. This structural analysis provides an explanation for the low affinity of the murine IFNβ/IFNAR2 interaction, compared to the high affinity human IFNβ/IFNAR2 interaction.

The murine IFNβ/IFNAR1 binary complex structure provides an important datapoint in the proposed model of human type-I IFN signaling. However, my lab and others have previously noted the “uniqueness” of type-I IFN families in different animals (10, 126–129). For example, the murine IFN system consists of 14 IFNαs (note that murine and human IFNα subtype designations have no bearing on their interspecies sequence and/or functional similarities), as well as IFNβ, IFNϵ, IFNκ, limitin (130), but do not encode an IFNω (126). Thus, it is necessary to ask if the murine IFNs and receptor proteins, as well as their biological outcomes, can be extrapolated to humans. From a structural biology perspective, the overall folds of murine (62) and human (5) IFNβ, which share 47% sequence identity, are almost identical (**Figure 6A**). The extracellular regions of human and murine IFNAR1 share 49% amino acid sequence identity and the structures of D1-D3 domains of murine and human IFNAR1s are also almost identical (119). These findings suggest the overall model proposed for the missing D4 domain in the human IFN/IFNAR2/IFNAR1 complex is plausible (**Figure 5**).

Despite similar overall receptor complex structures, the receptor binding properties of murine and human IFNβ are distinct. Human IFNβ binds to IFNAR1 and IFNAR2 with ~30nM and ~0.1nM *K*D values, respectively (28). However, in the mouse, IFNβ receptor affinities are “flipped” such that the IFNβ-IFNAR1 forms the high affinity interaction (*K*D ~10nM) and the IFNβ-IFNAR2 forms the low affinity interaction (*K*D ~1.7µM) (86). Structural comparisons of human and murine IFNβ reveal the AB loop of murine IFNβ, which forms a major part of the IFNAR2 site-1 binding site, exhibits a distinct structure compared to human IFNβ (**Figure 6**). In human IFNβ, the AB-loop arches toward the N-terminal end of helix-F, “over” helix F itself, where the loop connects to helix F by a disulfide bond. In contrast, the murine IFNβ AB-loop wraps “across” helix F where it would disrupt high affinity IFNAR2 interactions, as observed in the human IFNα/IFNAR2 crystal structure (**Figure 6B**). Interestingly, sequence alignments reveal the murine IFNAR2 receptor binding loops that contact the AB loop region of murine IFNβ are the same length as human IFNAR2. In addition, murine IFNαs bind with high affinity (*K*D ~1nM) to murine IFNAR2 (86). Thus, it is likely murine IFNAR2 receptor binding loops do not change their lengths, or grossly change their conformations, to accommodate the distinct murine IFNβ AB loop structure. Together, these structural observations provide an explanation for the low affinity of the murine IFNβ/IFNAR2 interaction, compared to the human IFNβ-IFNAR2 interaction. While this structural analysis is satisfying with respect to murine and human IFNβ, it highlights the many distinct properties of the murine IFNs, from structure to mechanism to *in vivo* outcomes, remain uncharacterized.

### Moving Forward

This review has focused on fundamental structural features of the three human IFN families, highlighting similar and unique features of each receptor complex. The ultimate goal of structural studies is to define mechanisms that can be used to discover optimal IFN therapeutics that harness the antiviral activity of the IFNs to improve human health (131). The importance of this goal is highlighted by the SARS-CoV-2 pandemic that is ravaging our society (72, 132–134). Based on the critical role that IFN – IFN receptor affinity plays in varying IFN activity (26, 120, 135), type-I and type-III IFNs with increased receptor affinity have been designed, yet they have not advanced into the clinic (79, 136, 137). Presumably because we still do not know the optimal design principles to create an optimal IFN therapeutic. Given that humans produce 20 different type-I/III IFNs in response to pathogens, the design may not be simple and might require the synergistic actions of both type-I and type-III IFNs. For example, type-I IFNβ and type-III IFNλ3 induced distinct anti-viral gene expression profiles with distinct kinetics on human hepatocytes (138). Specifically, high affinity IFNβ induced potent antiviral protection almost immediately (~2 h) after addition to cells that waned after ~48 h. In contrast, IFNλ3 antiviral activity was not observed until ~12 h after treatment, but was sustained for at least 72 h post-treatment (138). These data highlight the interplay of distinct receptor affinities and negative feedback mechanisms (139, 140), which synergistically control IFN-mediated antiviral signaling. Notably, type-III IFN signaling has been shown to be resistant to USP18-mediated negative feedback regulation, which potently regulates type-I IFN signaling (141). USP18 is induced by type-I and type-III IFNs, but specifically binds to the ICD of IFNAR2 and disrupts IFNα-mediated IFNAR1/IFNAR2 complex formation. These studies demonstrate that the anti-viral signaling cascade induced by type-I and type-III IFNs is very similar, yet multiple mechanisms can tailor the response for optimal functional outcomes, which include eliminating the virus and protecting the host. These studies, and more like them, are providing new design principles to further our quest for safe and efficacious IFNs with broad-spectrum antiviral activity.

## Author Contributions

MRW performed literature searches, made figures, and wrote the manuscript.

## Funding

This manuscript was funded in part by NIH grant R01 AI143554.

## Conflict of Interest

The author declares that the research was conducted in the absence of any commercial or financial relationships that could be construed as a potential conflict of interest.
